# The impact of the Quality and Outcomes Framework (QOF) on the recording of smoking targets in primary care medical records: cross-sectional analyses from The Health Improvement Network (THIN) database

**DOI:** 10.1186/1471-2458-12-329

**Published:** 2012-07-10

**Authors:** Jaspal S Taggar, Tim Coleman, Sarah Lewis, Lisa Szatkowski

**Affiliations:** 1 National School for Primary Care Research and UK Centre for Tobacco Control Studies, Division of Primary Care, University of Nottingham, Medical School, Queen’s Medical Centre, Nottingham, NG7 2UH, England; 2 Division of Epidemiology and Public Health, UK Centre for Tobacco Control Studies, Clinical Sciences Building, City Hospital Campus, Hucknall Road, Nottingham, NG5 1PB, England

**Keywords:** Smoking, Quality and Outcomes Framework (QOF), Targets

## Abstract

**Background:**

Smoking is a UK public health threat but GPs can be effective in helping patients to quit; consequently, the Quality and Outcomes Framework (QOF) incentivises the recording of smoking status and delivery of cessation advice in patients’ medical records. This study investigates the association between smoking-related QOF targets and such recording, and the factors which influence these clinical activities.

**Methods:**

For 2000 to 2008, using medical records in The Health Improvement Network (THIN) database, the annual proportions of i) patients who had a record of smoking status made in the previous 27 months and ii) current smokers recorded as receiving cessation advice in the previous 15 months were calculated. Then, for all patients at selected points before and after the QOF’s implementation, data on gender, age, Townsend score, and smoking-related morbidity were extracted. Multivariate logistic regression was used to investigate individual-level characteristics associated with the recording of smoking status and cessation advice.

**Results:**

Rapid increases in recording smoking status and advice occurred around the QOF’s introduction in April 2004. Subsequently, compliance to targets has been sustained, although rates of increase have slowed. By 2008 64.5% of patients aged 15+ had smoking status documented in the previous 27 months and 50.5% of current smokers had cessation advice recorded in the last 15 months. Adjusted odds ratios show that, both before and after the introduction of the QOF, those with chronic medical conditions, greater social deprivation and women were more likely to have a recent recording of smoking status or cessation advice. Since the QOF’s introduction, the strongest characteristic associated with recording activities was the presence of co-morbidity. An example of this was patients with COPD, who in 2008, were 15.38 (95% CI 13.70-17.27) times and 11.72 (95% CI 10.41-13.21) times more likely to have a record of smoking status and cessation advice, respectively.

**Conclusions:**

Rates of recording smoking status and cessation advice plateaued after large increases during the QOF’s introduction; however, recording remains most strongly associated with the presence of chronic disease as specified by the QOF, and suggests that incentivised targets have a direct effect on clinical behaviour.

## Background

Cigarette smoking is one of the most important modifiable risk factors in public health, accounting for over 100 000 deaths annually in the United Kingdom (UK) [1]. In addition to the significant mortality from smoking-related cancer and diseases of the cardiovascular and respiratory system [2], there is a considerable loss in quality of life from associated conditions such as asthma, osteoporosis and fractures [3]. The weekly cost to the NHS is estimated at over £50 million [4] and it is, therefore, unsurprising that the cessation of smoking is the single most cost-effective method for improving an individual’s health [5]. Primary care health professionals have a role in helping smokers to stop; when smoking is recorded prominently in medical records they are more likely to address this issue [6]. Amongst the interventions that they can use to promote smoking cessation, brief advice against smoking is a very simple intervention to deliver and is also known to be effective [7].

In April 2004, the Quality and Outcomes Framework (QOF), a comprehensive new contract which incentivised the provision of health care, was introduced for UK General Practitioners (GPs) [8]. The QOF identifies key domains and indicators across the spectrum of clinical activity and includes rewards for achieving targets set for recording, in medical records, patients’ smoking status and cessation advice given to smokers. Overall, it is estimated that remuneration from the QOF accounts for around 20% of practice income [9], of which smoking related targets contribute 8% [10].

Since the QOF’s introduction, targets for recording smoking status and cessation advice were revised in 2006 and again in 2008. For recording of smoking status, the QOF originally specified that, for patients without smoking-related morbidity, smoking status recorded at any time was sufficient for target ascertainment. However targets were different for patients with smoking-related morbidity; the 2004 QOF encouraged more frequent recording (every 15 months) of smoking status and advice giving to smokers with coronary heart disease, stroke or transient ischaemic attack (TIA), diabetes mellitus, chronic obstructive pulmonary disease (COPD), asthma and hypertension. In 2006, for the first time, recording smoking status in non-morbid patients was required periodically (every 27 months) rather than ‘ever’ to attract payment. In 2008, chronic kidney disease (CKD) and mental illness (schizophrenia, bipolar affective disorder and other psychoses) were added to the list of smoking-related conditions which required recording of smoking status and cessation advice every 15 months to attract remuneration; no changes were made to other smoking-related QOF targets. Table 1 summarises the smoking-related targets and subsequent changes since the QOFs introduction.

**Table 1:** The Quality and Outcomes Framework targets for the recording of smoking status and cessation advice in primary care medical records

	**2004**		**2006**		**2008**	
	**No co-morbidity**	**Co-morbidity†**	**No co-morbidity**	**Co-morbidity†**	**No co-morbidity**	**Co-morbidity**
**Smoking status**		The percentage of patients with any one or combination of these conditions whose notes record smoking status in the previous 15 months. Except those who have never smoked where the smoking status need only be recorded once since diagnosis.	The percentage of patients aged over 15 years whose notes	The percentage of patients with any one or combination of these conditions whose notes record smoking status in the previous 15 months. Except those who have never smoked where the smoking status need only be recorded once since diagnosis.	The percentage of patients aged over 15 years whose notes	The percentage of patients with any one or combination of these conditions whose notes record smoking status in the previous 15 months. Except those who have never smoked where the smoking status need only be recorded once since diagnosis.
**Cessation advice**	The smoking status of patients age 15 – 75 is recorded for at least 55 per cent of patients	The percentage of patients with any one or combination of these conditions who smoke whose notes contain a record that smoking cessation advice or referral to a specialist service, where available, has been offered within the previous 15 months.	record smoking status in the past 27 months, except those who have never smoked where smoking status need be recorded only once.	The percentage of patients with any one or combination of these conditions who smoke whose notes contain a record that smoking cessation advice or referral to a specialist service, where available, has been offered within the previous 15 months.	record smoking status in the past 27 months.	The percentage of patients with any one or combination of these conditions who smoke whose notes contain a record that smoking cessation advice or referral to a specialist service, where available, has been offered within the previous 15 months.

**†Co-morbidity 2004 & 2006** = coronary heart disease, diabetes mellitus, chronic obstructive pulmonary disease (COPD), transient ischaemic attack (TIA) or stroke, asthma, hypertension.

**Co-morbidity 2008** = As for 2006 co-morbidities + chronic kidney disease (CKD), Schizophrenia, bipolar disorder or other psychoses (“psychoses”).

Previous analyses using primary care data showed an increase in the incidence of recording smoking status and cessation advice to all smokers, with particularly high rates of recording in patients with smoking-related morbidity incentivised by the QOF’s [11]; however, these findings were limited by including data only until 2005. It is, therefore, not known if these increases have been sustained or how GPs have responded to the subsequent changes in smoking-related QOF targets. For example, the introduction of periodicity in recording smoking status for those without smoking-related morbidity in 2006 may have resulted in greater recording amongst non-morbid patients, reducing differences in ascertainment rates between patients with and without chronic diseases. To learn more about how GPs respond to targeted financial incentives, we investigated the changes in recording smoking status and cessation advice to smokers in primary care medical records, at time points before and after the QOF’s introduction and the factors influencing this.

## Method

### Setting and participants

We used electronic primary care medical records contained in The Health Improvement Network (THIN) database; this includes over six million patients’ records from 446 practices throughout the UK, and is broadly representative of the UK population in terms of patients’ demographic characteristics. The data were provided by Epidemiology and Pharmacology Information Core (EPIC), part of the CSD Medical Research Group (http://csdmruk.cegedim.com/about-us/about-us.html). This study was approved by the Leicestershire and Rutland Research Ethics Committee.

We performed cross sectional analyses of data collected from 2000 to 2008. For each year, patients aged 15+ who were registered in THIN on an index date of 1st April were identified. Outcome measures were defined as patients with a record of smoking status in the last 27 months and patients recorded as smokers with documented cessation advice in the last 15 months; patients were excluded from analysis if they had registered with a practice within the last three months, corresponding to the grace period GPs have to update the records of new patients (which includes the recording of smoking status). Annual trends in the outcome measures were determined for i) all eligible patients and ii) a restricted cohort of patients with at least one of the chronic illnesses specified by the QOF in 2008. To investigate the factors associated with the recording of smoking status and cessation advice, data were extracted from three index time points: April 2002 (before QOF), April 2004 (at introduction of QOF) and April 2008 (after QOF). Factors of interest were age, gender, socioeconomic status (SES) as determined by Townsend quintiles (quintile I representing the least deprived and quintile V the most deprived) and presence of any chronic illness (specified by the QOF in 2008). A summary variable ‘presence of at least one chronic condition named in QOF’ was also created. Missing data, present for Townsend quintiles, was identified and included as a separate category in the analyses.

### Statistical analyses

All analyses were performed using Stata version 10.0. Recording of smoking status and cessation advice were calculated as the annual percentages between 2000 and 2008 for patients with and without QOF-specified chronic illnesses and plotted graphically. The proportion with a recorded smoking status and proportion of recorded smokers receiving advice was also calculated separately according to patient characteristics (age, gender, SES and presence of QOF-defined chronic disease) at the three QOF-related time points. Logistic regression was used to investigate the independent associations between the patient characteristics and both outcome measures. Significantly associated factors were entered into a final multivariate model, which was adjusted for clustering by practice, to calculate adjusted odds ratios (OR) and corresponding confidence intervals (CI). Age and Townsend quintiles were included as categorical variables in the final model due to a non-linear association existing between these covariates and outcome measures (Likelihood Ratio Test (LRT) p < 0.001 for both covariates). Significance testing was performed using the Wald test or LRT for binary or categorical variables, respectively.

## Results

### Participant characteristics

A sample size of 1,998,631 participants was used in the analyses for 2002, which increased to 2,053,840 and 2,149,026 participants in 2004 and 2008, respectively. The mean (SD) age of patients in all analyses was 47.9 (19.0) years for patients with a record of smoking status and 44.6 (SD 16.1) years for patients with a record of advice against smoking.

**Figure 1:**
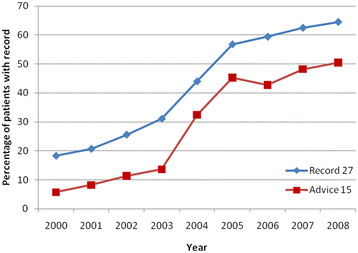
All patients from THIN with a record of smoking status within the last 27 months and cessation advice within the last 15 months from 2000 to 2008.

**Figure 2:**
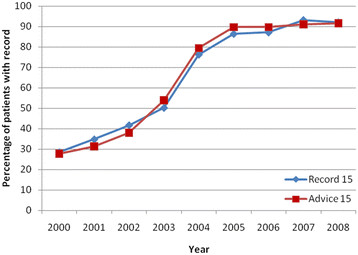
Patients from THIN with at least 1 chronic condition and having a record of smoking status or cessation advice within the last 15 months from 2000 to 2008.

Figure 1 illustrates the proportion of patients with a record of smoking status in the last 27 months and cessation advice to smokers in the last 15 months, annually between 2000 and 2008. Overall, a greater proportion of patients had a record of smoking status and cessation advice in 2008 as compared to 2004, and in 2004 compared to 2002. A substantial acceleration in recording of both smoking status and cessation advice was observed between 2003 and 2005, although rates of increase plateaued after 2006. Similar trends were observed for patients with at least one QOF-defined chronic condition (Figure 2), although the compliance to QOF targets was greater at every time point compared to non-morbid patients.

Characteristics of all study participants are presented in Table 2 and the adjusted ORs from the multivariate analyses are presented in Table 3. In 2008, 70.4% of women and 58.6% of men had their smoking status recorded and 57.1% of female and 44.6% of male smokers had a record of cessation advice. This was supported by the multivariate analyses, such that women in 2008, had a 71% increase in odds of having both a record of smoking status (OR 1.71, 95% CI 1.65-1.77, p < 0.001) and advice against smoking (OR 1.71, 95% CI 1.66-1.77, p < 0.001). Based on a qualitative comparison of ORs and CIs, the strength of these associations appears similar in 2004 and 2008, but stronger than in 2002, suggesting that gender has had a greater influence on both clinical activities since the introduction of the QOF.

**Table 2:** Characteristics of patients with a record of smoking status and cessation advice in 2002, 2004 and 2008

	**2002**				**2004**				**2008**			
	Patients with smoking status recorded in last 27 months		Current smokers with advice recorded in last 15 months		Patients with smoking status recorded in last 27 months		Current smokers with advice recorded in last 15 months		Patients with smoking status recorded in last 27 months		Current smokers with advice recorded in last 15 months	
	Denominator	%	Denominator	%	Denominator	%	Denominator	%	Denominator	%	Denominator	%
All patients	1,998,631	25.6	421,358	11.3	2,053,840	44.0	453,646	32.4	2,149,026	64.5	463,457	50.5
Age†												
15-24	241,936	18.6	36,468	13.7	261,188	31.6	47,638	36.4	298,969	53.9	61,375	47.5
25-34	296,333	22.3	85,289	10.1	286,938	36.7	89,143	26.6	282,456	55.9	89,525	40.9
35-44	385,447	22.9	93,873	10.7	394,638	37.4	104,037	27.1	395,272	57.3	105,830	43.8
45-54	345,378	26.3	80,974	11.7	350,101	42.7	85,565	31.5	373,439	62.6	88,247	50.5
55-64	299,840	30.8	65,769	12.4	321,177	50.9	69,893	37.7	341,211	70.3	67,240	59.4
65-74	224,454	33.0	37,606	12.4	232,115	59.8	36,970	44.3	239,648	80.3	33,928	71.8
≥ 75	205,243	27.2	21,379	7.9	207,683	56.3	20,400	40.6	218,031	80.4	17,312	74.9
Gender†												
Male	988,459	21.5	211,056	10.1	1,019,442	37.7	230,573	28.4	1,064,916	58.6	247,672	44.6
Female	1,010,172	29.6	210,302	12.5	1,034,398	50.2	223,073	36.4	1,084,110	70.4	215,785	57.1
Townsend category†												
I	450,110	24.9	68,892	10.7	469,438	42.4	71,931	28.9	495,833	62.1	67,801	47.0
II	382,371	26.1	68,820	11.3	395,531	44.2	72,939	30.0	418.070	64.6	71,872	49.8
III	360,749	26.5	79,603	11.9	372,521	45.0	86,009	32.8	396,779	65.5	88,699	50.9
IV	309,129	27.5	81,939	12.4	318,962	46.2	90,016	34.2	340,826	66.5	96,564	51.9
V	211,144	26.4	65,709	11.9	218,173	45.3	73,396	34.6	233,040	67.8	81,531	53.0
Missing	285,128	22.5	56,395	9.2	279,215	41.5	59,355	33.1	264,478	62.2	56,990	48.7
Chronic conditions†												
No CHD	1,907,513	24.3	405,044	10.9	1,955,671	42.0	437,596	31.0	2,050,655	63.2	449,704	49.2
CHD	91,118	52.8	16,314	20.9	98,169	84.1	16,050	68.8	98,371	93.4	13,753	92.9
No Stroke / TIA	1,962,125	25.4	414,504	11.3	2,014,161	43.5	446,817	31.9	2,106,472	64.0	457,201	49.9
Stroke / TIA	36,506	38.3	6,854	14.7	39,679	72.0	6,829	59.3	42,554	89.1	6,256	91.9
No CKD	1,998,236	25.6	421,296	11.3	2,052,590	44.0	453,469	32.4	2,046,855	63.4	453,837	49.7
CKD	395	37.5	62	8.1	1,250	55.8	177	42.4	102,171	88.4	9,620	87.0
No Psychoses	1,988,078	25.5	417,503	11.3	2,041,506	44.0	448,735	32.3	2,134,016	64.4	457,314	50.3
Psychoses	10,553	29.4	3,855	12.6	12,334	52.1	4,911	37.9	15,010	83.0	6,143	64.7
No COPD	1,969,089	25.3	410,412	11.1	2,020,769	43.4	441,983	31.5	2,106,205	63.9	449,485	49.1
COPD	29,542	49.8	10,946	20.1	33,071	82.3	11,663	66.4	42,821	98.1	13,972	95.4
No Hypertension	1,769,912	23.5	382,822	10.9	1,777,210	39.8	410,782	29.9	1,818,626	60.2	419,611	46.2
Hypertension	228,719	41.8	38,536	16.0	276,630	71.7	42,864	55.5	330,400	88.4	43,846	91.5
No DM	1,970,857	25.1	416,325	11.2	2,005,687	43.0	445,475	31.6	2,055,686	63.1	449,206	49.1
DM	27,774	59.4	5,033	20.3	48,153	88.1	8,171	71.9	93,340	96.2	14,251	95.1
No Asthma	468,235	24.7	398,582	10.9	1,939,817	42.3	427,707	30.6	2,029,919	62.9	440,467	48.4
Asthma	43,891	43.0	22,776	19.3	114,023	72.5	25,939	60.9	119,107	92.3	22,990	90.9
No ≥ 1 CC	336,405	21.2	339,714	9.9	1,579,698	35.7	363,324	26.2	1,584,842	55.7	370,482	40.8
≥ 1 CC	175,721	42.7	81,644	17.3	474,142	71.9	90,322	57.1	564,184	89.3	92,975	89.0

[† = % values calculated as row percentages].

**Table 3:** Multiple logistic regression analyses for the recording of smoking status and cessation advice in 2002, 2004 and 2008

	**2002**				**2004**				**2008**			
	**Record 27**		**Advice 15**		**Record 27**		**Advice 15**		**Record 27**		**Advice 15**	
N	1,998,631		421,358		2,053,840		453,646		2,149,026		463,457	
	**AOR**	**P**	**AOR**	**P**	**AOR**	**P**	**AOR**	**P**	**AOR**	**p**	**AOR**	**P**
	**(95% CI)**		**(95% CI)**		**(95% CI)**		**(95% CI)**		**(95% CI)**		**(95% CI)**	
Age												
15-24	1	<0.001	1	<0.001	1	<0.001	1	<0.001	1	<0.001	1	<0.001
25-34	1.25		0.73		1.25		0.64		1.06		0.78	
	(1.19-1.31)		(0.69-0.77)		(1.20-1.29)		(0.62-0.67)		(1.02-1.10)		(0.75-0.81)	
35-44	1.26		0.77		1.24		0.65		1.08		0.82	
	(1.19-1.31)		(0.72-0.82)		(1.19-1.29)		(0.62-0.68)		(1.04-1.13)		(0.79-0.85)	
45-54	1.40		0.79		1.40		0.72		1.21		0.90	
	(1.33-1.47)		(0.73-0.85)		(1.34-1.46)		(0.68-0.75)		(1.15-1.26)		(0.86-0.94)	
55-64	1.51		0.75		1.62		0.80		1.38		0.98	
	(1.43-1.60)		(0.69-0.81)		(1.54-1.70)		(0.76-0.84)		(1.32-1.45)		(0.94-1.02)	
65-74	1.41		0.66		1.84		0.85		1.80		1.25	
	(1.32-1.50)		(0.60-0.72)		(1.73-1.96)		(0.77-0.93)		(1.68-1.92)		(1.16-1.35)	
≥ 75	0.93		0.37		1.29		0.62		1.28		1.02	
	(0.85-1.03)		(0.32-0.43)		(1.20-1.40)		(0.55-0.71)		(1.19-1.38)		(0.92-1.13)	
Gender												
Male	1	n/a	1	n/a	1	n/a	1	n/a	1	n/a	1	n/a
Female	1.54	<0.001	1.27	<0.001	1.68	<0.001	1.47	<0.001	1.71	<0.001	1.71	<0.001
	(1.48-1.60)		(1.22-1.31)		(1.63-1.73)		(1.43-1.50)		(1.65-1.77)		(1.66-1.77)	
Townsend Category												
I	1	<0.001	1	<0.001	1	<0.001	1	<0.001	1	<0.001	1	<0.001
II	1.06		1.06		1.06		1.04		1.10		1.11	
	(1.00-1.17)		(0.98-1.14)		(1.01-1.10)		(0.98-1.10)		(1.06-1.14)		(1.06-1.15)	
III	1.08		1.10		1.11		1.18		1.18		1.17	
	(1.04-1.25)		(1.00-1.21)		(1.04-1.18)		(1.10-1.26)		(1.11-1.25)		(1.10-1.25)	
IV	1.14		1.14		1.16		1.23		1.25		1.19	
	(1.06-1.28)		(1.01-1.29)		(1.08-1.25)		(1.14-1.33)		(1.16-1.34)		(1.12-1.26)	
V	1.07		1.06		1.12		1.21		1.35		1.20	
	(0.93-1.23)		(0.90-1.26)		(1.00-1.26)		(1.09-1.35)		(1.21-1.49)		(1.10-1.30)	
Missing	0.90		0.83		0.99		1.20		1.05		1.06	
	(0.70-1.15)		(0.59-1.17)		(0.85-1.15)		(0.96-1.48)		(0.92-1.21)		(0.90-1.26)	
Chronic Conditions												
CHD	3.02	<0.001	2.16	<0.001	5.12	<0.001	3.66	<0.001	4.16	<0.001	7.14	<0.001
	(2.78-3.27)		(1.92-2.43)		(4.72-5.56)		(3.37-3.97)		(3.77-4.59)		(6.43-7.93)	
Stroke / TIA	1.28	<0.001	1.23	<0.001	1.74	<0.001	1.82	<0.001	1.83	<0.001	4.56	<0.001
	(1.22-1.33)		(1.11-1.36)		(1.67-1.85)		(1.68-1.96)		(1.71-1.95)		(4.02-5.17)	
CKD	1.07	0.662	0.58	0.175	0.73	0.305	0.78	0.515	1.42	<0.001	1.76	<0.001
	(0.78-1.47)		(0.26-1.28)		(0.40-1.33)		(0.37-1.66)		(1.33-1.52)		(1.61-1.91)	
Psychoses	1.12	0.027	1.11	0.072	1.28	<0.001	1.26	<0.001	2.51	<0.001	1.77	<0.001
	(1.01-1.24)		(0.99-1.25)		(1.20-1.37)		(1.17-1.36)		(2.35-2.69)		(1.64-1.91)	
COPD	2.03	<0.001	1.81	<0.001	3.37	<0.001	2.72	<0.001	15.38	<0.001	11.72	<0.001
	(1.90-2.17)		(1.65-1.99)		(3.11-3.65)		(2.50-2.96)		(13.70-17.27)		(10.41-13.21)	
Hypertension	1.87	<0.001	1.51	<0.001	2.58	<0.001	2.29	<0.001	2.95	<0.001	8.49	<0.001
	(1.77-1.97)		(1.41-1.61)		(2.45-2.72)		(2.17-2.42)		(2.76-3.15)		(7.74-9.32)	
Diabetes	3.20	<0.001	1.74	<0.001	6.23	<0.001	3.99	<0.001	7.85	<0.001	12.00	<0.001
	(2.82-3.63)		(1.41-2.15)		(5.61-6.93)		(3.58-4.45)		(6.95-8.86)		(10.66-13.52)	
Asthma	2.05	<0.001	1.74	<0.001	3.34	<0.001	3.14	<0.001	6.70	<0.001	10.26	<0.001
	(1.93-2.18)		(1.63-1.84)		(3.15-3.54)		(2.93-3.36)		(6.27-7.17)		(9.37-11.23)	
≥ 1 CC	2.76	<0.001	2.11	<0.001	4.16	<0.001	3.70	<0.001	5.80	<0.001	10.83	<0.001
	(2.63-2.89)		(1.99-2.24)		(3.98-4.34)		(3.51-3.90)		(5.49-6.14)		(10.16-11.53)	

[AOR = Adjusted Odds Ratio; Record 27 = Patients with a record of smoking status within the previous 27 months; Advice 15 = Patients who are current smokers with a record of advice against smoking within the previous 15 months; adjustments made for age category, gender, Townsend category and presence of chronic illness; P = p value: LRT used for age and Townsend quintiles, Wald test used for other covariates].

There were more patients with a record of smoking status with advancing age such that in 2008 80.4% of patients over 75 years had a record of smoking status as compared to 53.9% of those aged 15–24 years. In contrast, a u-shaped relationship between recording of cessation advice and age was observed; in 2008, 47.5% of 15–24 year olds had a record of cessation advice, which decreased to 40.9% in 25–44 year olds before increasing through other categories to 74.9% in those aged over 75. The effect of age was sustained and independently significant in the multivariate analyses for both recording of smoking status and cessation advice (LRT for categories p < 0.001).

There was a greater recording of smoking status and cessation advice with advancing Townsend score (greater deprivation); this was most apparent in 2008, when 67.8% and 53.0% of patients had smoking status and cessation advice recorded in the most deprived quintile, respectively. Multivariate analyses for 2008 showed that patients with greater deprivation were 35% more likely to have smoking status recorded (OR 1.35, 95% CI 1.21-1.49, p < 0.001) and 20% more likely to have cessation advice recorded (OR 1.20, 95% CI 1.10-1.30, p < 0.001), than those least deprived.

Generally, in all years (with the exceptions of CKD in 2002 and 2004, and psychoses in 2002), those with chronic conditions were more likely to have a record of smoking status and cessation advice. This was also evident for patients with a history of at least one QOF-defined chronic condition. Mostly, the ORs describing the association between morbidities and target compliance were greatest in 2008, and in 2004 they were higher compared to those in 2002. With the exception of a past history of CKD and psychoses, all chronic conditions were associated with a substantial increase in the odds of having a record of smoking status and cessation advice between 2004 and 2008. An example of this was patients with COPD, who in 2004 were 3.37 times more likely to have their smoking status recorded (OR 3.37, 95% CI 3.11-3.65, p < 0.001), compared to those without co-morbidity, increasing to 15.38 times more likely in 2008 (OR 15.38, 95% CI 13.70-17.27, p < 0.001). For the recording of cessation advice, COPD patients were 2.72 times more likely to have this recorded in 2004 (OR 2.72, 95% CI 2.50-2.96, p < 0.001) which increased to 11.72 times more likely in 2008 (OR 11.72, 95% CI 10.41-13.21, p < 0.001). Similar trends were observed when the presence of at least one chronic disease was analysed separately.

## Discussion

### Summary of main findings

This study found that substantial increases in the recording of smoking status and cessation advice in primary care medical records, after the introduction of the QOF, have been sustained. Similar trends in recording for patients with and without QOF-specified co-morbidities were observed, with rates of compliance greater for those with chronic diseases throughout the whole study period. Patients with QOF-defined chronic disease, women and those with greater social deprivation were independently more likely to have both a record of smoking status and cessation advice. The presence of co-morbidity had the greatest influence on these activities as evidenced by substantial increases in ORs describing the association between chronic illness and target compliance after the QOF’s introduction. In 2008, patients with at least one chronic disease were six-times more likely to have a record of smoking status, and those who smoked were 11-times more likely to have cessation advice recorded. Furthermore, only in 2008, after CKD and mental illness had been included in the list of QOF-incentivised chronic conditions, were positive associations observed between recording and the possession of these diseases.

### Strengths and limitations

Limitations of this study include the inherent weaknesses associated with using a cross sectional study design, such as the inability to establish a causal relationship between the QOF and recording activities. By taking repeated cross sectional samples at time points before and after the QOFs introduction, the temporal relationship between recording patterns and QOF targets were more appreciable. Confounding by other anti-smoking interventions at around the time of the QOFs introduction may have influenced our results. Comprehensive smokefree legislation covering all enclosed public places (including bars and restaurants) was introduced in Scotland in 2006 and in the rest of the UK in 2007. During this time more smokers attempted to quit [12] and may have presented to GPs for cessation support [13]; this may have affected GPs’ recording of smoking data and thus any changes observed during this time period may not be attributable to the QOF. However, the trends in recording smoking status and cessation advice plateaued during this period in our study, which is inconsistent with this hypothesis. Conversely, stronger associations between individual level factors, such as the presence of co-morbidity, and target recording were observed in 2008, suggesting that these factors were more influential on the recording behaviour of GPs. The outcome measures used in this study are not rare, so another limitation is that the reported OR’s are likely to represent an over-estimate of risk ratios.

A major strength of this study was its large sample size; precise estimates were calculated as reflected by the narrow CIs for the ORs. Differences in smoking prevalence according to age, gender and SES have been described previously (13) and the methodological approach used enabled the effects of these confounders to be adjusted for. However, there is the potential for residual confounding in this study; regional differences in smoking prevalence, marital status and ethnicity were not accounted for and may influence the recording of smoking targets [14,15].

### Comparison with existing literature

Consistency with previous research suggests that our study findings are valid. An acceleration in the recording of smoking status and cessation advice during the QOF’s introduction were previously described by Coleman et al [11], and we found similar trends in recording activity. Previous findings were limited by the restriction of analyses up until 2005 but our study reports trends until 2008; we found a plateau in the recording of smoking status and advice against smoking after 2006. An implication of this may be that current targets are no longer promoting change in this clinical behaviour and that new or reworded targets may be required to stimulate further improvements in smoking management. An analysis from the QRESEARCH database investigated the trends in smoking prevalence between 2001 and 2007 [16], and also reported a greater recording of smoking status and cessation advice in medical records for women. These findings may reflect the higher consultation rates in primary care of women [17] who, therefore, are more likely to have smoking targets recorded at routine clinical enquiry. We found social deprivation to be associated with the recording of smoking targets, such that in 2008, patients with greater deprivation were more likely to have both a record of smoking status and cessation advice. Although the QOF smoking targets do not make reference to social deprivation, this finding may reflect higher consultation rates for patients with multiple co-morbidities, older age or greater social deprivation [18].

This study is unique as it investigated the impact of morbidity on GP recording activities. To date, evidence evaluating the impact of morbidity on the smoking specific QOF targets is limited [11]. The presence of co-morbidity was most strongly associated with the recording of QOF targets, and this was greatest in 2008. Furthermore, conditions such as CKD and mental illness were only recently included in QOF targets; significant associations for these conditions were only observed in the later years of analysis, and suggests the specific wording within QOF targets is influential on clinical behaviour.

The observed quality of primary health care delivered, as defined by the compliance to QOF targets, has substantially improved for many indicators [19-22] since the QOF’s introduction. However, there have been few specific studies examining the impact of the QOF on the smoking related targets [23,24]. A study of data from the Scottish Programme for Improving Clinical Effectiveness in Primary Care (SPICE-PC) investigated the recording of risk factors for five QOF incentivised chronic diseases, which included the recording of smoking status [23]. Generally, the recording of risk factors was greater for targeted disease than for the general population, but Sutton and colleagues reported a dramatic increase in the recording of incentivised risk factors at the time of the QOFs introduction. This was comparable to a more natural increase in the recording of non-incentivised risk factors, and suggests that GPs were more responsive to the specific targets set within each co-morbidity. A recent analysis of GPRD data reported similar findings, although Doran and colleagues also found detrimental effects on the long-term achievement of non-incentivised targets, with lower rates of compliance being observed for these than expected [24].

Current QOF smoking targets are based on recording smoking status and providing brief cessation advice to those with co-morbidity, and do not reward the provision of other smoking cessation therapies. Indeed, these may not translate into delivered quality of care and successful smoking cessation attempts. A retrospective analysis of Scottish registry data after the introduction of the QOF showed the quality of care actually delivered to patients with incentivised chronic illness was worse in those with greater deprivation [25]. Furthermore, a recent comparison made between the recording of advice against smoking in patient notes and the predicted recall of cessation advice by patients in Primary Care Trust (PCT) surveys [26], found that patient recall of cessation advice was lower than that recorded in 2005 and 2008. Therefore, caution needs to be taken in attributing our study findings to suggest the provision of effective advice against smoking [10]. To be effective, incentive-based targets need to be regularly revised to ensure that they stimulate improvements in health care and clinical outcomes [27]. Current targets could be expanded to remunerate the provision of cessation advice to all patients and not just those with co-morbidity. Furthermore, additional targets may be required that encourage the provision of other smoking cessation therapies and successful quit attempts.

## Conclusions and recommendations

The recording of smoking related QOF targets in patient’s medical records has substantially increased since the introduction of the QOF although rates of compliance have plateaued in recent years; observed increases are most evident in those patients for whom such recording is remunerated. The effects of compliance with these targets on patient’s smoking behaviour remains unknown; research is required to investigate whether target achievement translates into smoking cessation. To ensure that QOF targets not only continue to influence recording, but also positively influence smoking behaviour, amendments in their wording may be required that focus on the incentivised provision of effective smoking cessation therapies.

## Competing interests

The authors declare that they have no competing interests

## Author contributions

All authors contributed to the design of this research study. JST produced the first draft of the manuscript and all authors have contributed to subsequent revisions and preparation of the final report. All authors read and approved the final manuscript.

## Pre-publication history

The pre-publication history for this paper can be accessed here:

http://www.biomedcentral.com/1471-2458/12/329/prepub
